# Effect of exercise‐induced bronchoconstriction on the configuration of the maximal expiratory flow‐volume curve in adults with asthma

**DOI:** 10.14814/phy2.15614

**Published:** 2023-02-23

**Authors:** Oksana Klimenko, Peter Luu, Paolo Dominelli, Nathan Noggle, Gregory Petrics, Hans Christian Haverkamp

**Affiliations:** ^1^ Department of Nutrition and Exercise Physiology Washington State University‐Spokane Health Sciences, Elson S. Floyd College of Medicine Spokane Washington USA; ^2^ Department of Kinesiology and Health Sciences University of Waterloo Waterloo Ontario Canada; ^3^ Department of Mathematics Northern Vermont University‐Johnson Johnson Vermont USA

**Keywords:** asthma, bronchoconstriction, exercise, flow‐volume curve, maximal expiration

## Abstract

We determined the effect of exercise‐induced bronchoconstriction (EIB) on the shape of the maximal expiratory flow‐volume (MEFV) curve in asthmatic adults. The slope‐ratio index (SR) was used to quantitate the shape of the MEFV curve. We hypothesized that EIB would be accompanied by increases in SR and thus increased curvilinearity of the MEFV curve. Adult asthmatic ( *n*  = 10) and non‐asthmatic control subjects ( *n*  = 9) cycled for 6–8 min at 85% of peak power. Following exercise, subjects remained on the ergometer and performed a maximal forced exhalation every 2 min for a total 20 min. In each MEFV curve, the slope‐ratio index (SR) was calculated in 1% volume increments beginning at peak expiratory flow (PEF) and ending at 20% of forced vital capacity (FVC). Baseline spirometry was lower in asthmatics compared to control subjects (FEV_1_% predicted, 89.1 ± 14.3 vs. 96.5 ± 12.2% [SD] in asthma vs. control; *p*  < 0.05). In asthmatic subjects, post‐exercise FEV_1_ decreased by 29.9 ± 13.2% from baseline (3.48 ± 0.74 and 2.24 ± 0.59 [SD] L for baseline and post‐exercise nadir; *p*  < 0.001). At baseline and at all timepoints after exercise, average SR between 80 and 20% of FVC was larger in asthmatic than control subjects (1.48 ± 0.02 vs. 1.23 ± 0.02 [SD] for asthma vs. control; *p* < 0.005). This averaged SR did not change after exercise in either subject group. In contrast, post‐exercise SR between PEF and 75% of FVC was increased from baseline in subjects with asthma, suggesting that airway caliber heterogeneity increases with EIB. These findings suggest that the SR‐index might provide useful information on the physiology of acute airway narrowing that complements traditional spirometric measures.

## Graphical abstract

We applied the slope‐ratio (SR) analysis to determine the effect of exercise‐induced bronchoconstriction (EIB) on the shape of the maximal expiratory flow‐volume curve in asthmatic adults. Post‐exercise SR between peak expiratory flow and 75% of vital capacity was increased from baseline in subjects with asthma, suggesting that airway caliber heterogeneity increases with EIB.

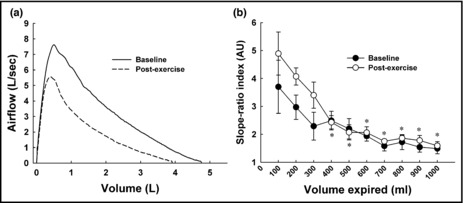

**New and noteworthy**

In a group of adults with asthma and a group of control subjects, we calculated the slope‐ratio index (SR) to analyze the curvilinearity of the maximal expiratory flow‐volume curve before and serially after exercise. In the asthmatic subjects, exercise‐induced bronchoconstriction was associated with increased SR at high lung volumes, and thus increased curvilinearity, of the maximal expiratory flow‐volume curve. The increased curvilinearity became apparent within a few minutes after exercise and it was still increased at 20 minutes after exercise. These findings suggest that the SR‐index might complement traditional measures of expiratory flow by providing a simple, inexpensive technique for characterizing the effects of acute bronchoconstriction on ventilation heterogeneity in adults with asthma.

## 1 INTRODUCTION

Exercise‐induced bronchoconstriction (EIB) refers to airway narrowing that is stimulated by whole‐body exercise. Typically, EIB is diagnosed by measuring spirometry before and serially after a high‐intensity exercise bout of moderate duration (6–8 min) (Parsons et al., 2013). The presence and severity of EIB is defined by the reduction in forced expiratory volume in 1 second (FEV_1_) after exercise, where a decrease of at least 10% from baseline is normally required for a positive diagnosis (Parsons et al., 2013). Although airway narrowing can occur during exercise in the asthmatic (Crimi et al., 2002; Klansky et al., 2016; Milanese et al., 2009), the relative brevity and high intensity of the standard 6–8 min protocol prevents such narrowing from occurring until after exercise cessation. Peak EIB occurs 5–15 min after exercise, and thereafter resolves on its own within approximately 1 h (Hallstrand et al., 2005).

**FIGURE 1:**
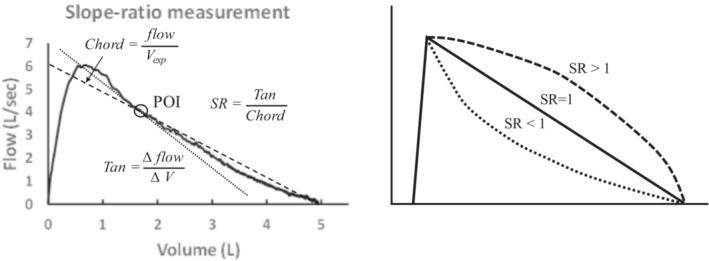
(a) Illustration of method for calculating slope‐ratio index. The maximal expiratory flow‐volume curve is from a female with asthma. (b) Illustration of slope‐ratio values for three idealized maximal expiratory flow‐volume curves. POI, point of interest; SR, slope‐ratio; V_exp_, volume remaining to be expired.

In addition to the discrete variables derived from spirometry (i.e., forced vital capacity, FEV_1_, mid forced expiratory flows), the overall shape of the maximal expiratory flow‐volume curve (MEFV) itself provides information on airway and lung (patho) physiology. For example, in chronic obstructive pulmonary disease (COPD), the MEFV is convex toward the volume axis (“scooped”) due to unequal airway narrowing and the loss of lung elastic recoil (Johns et al., 2014). In asthma, the MEFV curve can also exhibit a scooped appearance despite preserved lung elasticity (Crimi et al., 2002; Mead, 1978; Rossman et al., 2022). In both asthma and COPD, the nonlinear MEFV curve is thought to result from heterogeneous time constants for airway emptying due to unequal airway narrowing and differences in regional lung unit elastic recoil (Mead, 1978). Several approaches have been developed to quantitate the phenotype of the MEFV curve (Hoesterey et al., 2019). One measurement, known as the slope‐ratio index (SR), quantifies the degree of curvature at any point along the forced expiration (see Figure 1) (Mead, 1978). Previous work suggested an unchanged SR in asthmatics during an episode of EIB (Mead, 1978). Similarly, SR was also unchanged following a eucapnic voluntary hyperpnea challenge in a group of adults with asthma (Dominelli et al., 2016). In contrast, SR was altered following histamine inhalation in asthmatics (O'Donnel et al., 1986).

In this study, we determined the effect of EIB on the configuration of the MEFV curve in adults with asthma. In addition, a control group of non‐asthmatic subjects completed the studies, allowing for greater insight into the physiology of airway narrowing with exercise. In the previous report of unchanged SR after a EVH challenge in adults with asthma, the SR analysis was calculated between 80% and 20% of vital capacity (Dominelli et al., 2016). Also, subjects performed only one post‐EVH maximal forced expiration at 10 min after the challenge; the SR analysis was limited to this single post‐challenge MEFV curve. In this study, we analyzed SR beginning at peak expiratory flow and also report SR at multiple post‐exercise timepoints. We hypothesized that EIB would be accompanied by increased curvilinearity of the MEFV curve. Our findings suggest that the non‐invasive, simple SR analysis could be a useful approach for identifying worsened airway caliber heterogeneity during acute periods of airway narrowing in asthmatic adults.

## 2 METHODS

### 2.1 Subjects

Adult male and female asthmatic (*n* = 10) and non‐asthmatic (*n* = 9) subjects were recruited through posted advertisements in the local community. All subjects were non‐smokers between the ages of 18–45 years. Subjects were fully informed of the procedures, risks, and benefits of the study, and all subjects signed the informed consent document. This study was approved by the Institutional Review Board for research involving human subjects, and it was conducted in compliance with the Declaration of Helsinki, except for registration in a database.

All subjects had a negative history for cardiovascular disease and other chronic illness (excepting asthma), and an absence of respiratory infection during the 6‐weeks prior to participation. Subjects using oral or inhaled corticosteroids were excluded from participation. Subjects were instructed to refrain from using inhaled short‐acting β_2_‐agonist for 12 h prior to each study and from ingesting any products containing caffeine for 8 h prior to each study. Exercise was avoided for 8 h prior to each lab visit.

### 2.2 Experimental design

Two preliminary visits were completed to determine baseline characteristics and study eligibility. During the first visit, spirometry was completed before and 15‐min after four actuations of a fast‐acting inhaled bronchodilator (4 × 90 μg albuterol sulfate and using a spacer) (bronchodilator responsiveness [BD_res_]). During the second visit, subjects completed a maximal incremental exercise test‐to‐exhaustion on a cycle ergometer, preceded and followed by serial spirometry. The incremental exercise test was used to determine the exercise workload for the experimental trial and served to minimize the influence of learning effects on the experimental trial. After the two preliminary visits, all subjects completed one experimental study in the laboratory. Baseline spirometry was performed upon arrival to the lab. Then, while seated on the cycle ergometer, a series of maximum forced exhalations were performed. Following resting data collection, subjects exercised for 6–8 min at 80%–85% of their peak workload. The workload was applied gradually over 60 s and subjects self‐selected their cadence (minimum cadence, 60 rev·min^−1^). After exercise, subjects stopped pedaling and remained seated on the ergometer and with the mouthpiece and nose clips in place. Subjects performed a single maximum forced expiration every 2 min for a total of 20 min after the exercise; 10 maneuvers in total. Other than the maximum forced expirations, subjects were instructed to breathe spontaneously during the 20‐min post‐exercise period. To qualify as an asthmatic for this study, at least two out of the 10 post‐exercise FEV_1_ values had to be decreased by >12% from baseline. Participants breathed ambient air during the experimental study (temperature, 23.6 ± 1.9°C; atmospheric pressure, 742.5 ± 4.2 mmHg; relative humidity, 31.8 ± 16.2%).

### 2.3 Spirometry

Spirometry was completed in the seated, upright position according to recommendations by the American Thoracic Society and European Respiratory Society (Miller et al., 2005). During each measurement, subjects performed forced vital capacity maneuvers for determination of peak expiratory flow (PEF), forced vital capacity (FVC), FEV_1_, and forced expiratory flow between 25% and 75% of FVC (FEF_25‐75%_). Predicted values are from Quanjer (Quanjer et al., 2012).

### 2.4 Exercise apparatus and measurement instruments

Exercise was completed on a magnetically braked cycle ergometer (Velotron). Subjects breathed through a two‐way, non‐rebreathing valve (Hans‐Rudolph) with nose clips in place. Separate pneumotachographs (Hans‐Rudolph) were used to determine inspiratory and expiratory airflow. Separate oxygen and carbon dioxide gas analyzers (AEI‐Technologies) were used to analyze expired gases. A 16‐channel analog‐to‐digital data acquisition system (ADinstruments) interfaced with a laptop computer was used to collect resting and exercise data. Inspired and expired airflow and expired gases were continuously collected for calculation of oxygen consumption (${\overset{\cdot }{V}}_{O2}$) and carbon dioxide production (${\overset{\cdot }{V}}_{\mathrm{CO}2}$), ventilation (${\overset{\cdot }{V}}_{E}$), tidal volume (VT), and breathing frequency (fb).

### 2.5 Maximal expiratory flow‐volume curve analyses

Several analyses were performed on the pre‐ and post‐exercise MEFV curves. Forced vital capacity, FEV_1_, and PEF were determined in each curve. Area under the curve was also calculated in all MEFV curves. The slope‐ratio (SR) analysis, originally described by Mead (Mead, 1978), was performed on each MEFV curve. Briefly, to conduct the SR analysis, a MEFV curve is partitioned into a number of points of interest along the volume axis (Figure 1). At each point of interest, two lines are generated: a line of tangency and a chord line passing through the point of interest and residual volume. At each point of interest, a SR is calculated by dividing the tangent slope by the chord slope; this results in a unitless number that describes the shape of the MEFV curve at each point. As previously described (Dominelli et al., 2016), linear MEFV curves result in a SR of 1.0; curves that are convex to the volume axis (“scooped”) result in a SR greater than 1.0; curves that are concave to the volume axis result in a SR less than 1.0.

In most previous studies, SR calculations were confined to lung volumes between 80% and 20% of vital capacity. On the one hand, this approach limits the analysis to the effort‐independent portion of the forced exhalation. However, it fails to capture the lung volumes between PEF and the first SR measurement at 80% vital capacity. In persons with asthma, MEFV curves are often curvilinear at such high lung volumes, therefore providing further insight into the physiology of maximal airflow. For this reason, we analyzed the MEFV curves beginning at PEF and ending at 20% of FVC. We used Microsoft Excel to perform the SR analysis. Each curve was partitioned into 100 equal volume segments (i.e., 1%–100% FVC), and SR was calculated at each 1.0% volume segment between PEF and 20% of FVC. The tangent lines were generated by connecting the two points immediately preceding and following each point of interest. During preliminary analyses, we performed the SR analysis using several different volume increments (40–200 mL) and compared the findings. While larger volume increments result in reduced SR variability, they also fail to capture undulations in the MEFV curve that might provide insight into the physiology of airway emptying. In particular, small undulations, or small peaks and valleys, in the MEFV curve may indicate non‐homogeneous airway emptying or rapid movement of the equal pressure point to more distal airways (Bhatt et al., 2019). Using this reasoning, SR variability represents a useful physiologic signal rather than random regions with low signal‐to‐noise. Thus, despite the increased variability of the SR values resulting from the use of small volume increments, the tangent lines were determined using the volumes immediately preceding and following each point of interest; the two datapoints were 1% below and 1% above the point of interest. In our subjects, the average volume increment corresponding to 1% of FVC was 45 ± 12 and 41 ± 8 mL for control and asthmatic subjects, respectively. This volume increment is smaller than those used in previous analyses.

### 2.6 Statistical analysis

Independent t‐tests were used to compare baseline descriptive characteristics between control and asthma subjects. A two‐factor [**GROUP** (control and asthma) × **TIME** (baseline and post‐exercise timepoints)] repeated measures analysis of variance was used to analyze the post‐exercise responses. When significant main effects were found, pairwise comparisons using Bonferroni‐corrected p‐values were conducted to determine significance of the comparisons. Linear regression was used to analyze relationships between selected variables. Tabular data are presented as mean ± standard deviation. Data in figures are presented as mean ± standard error of mean. Significance was set at *α* ≤ 0.05. The statistical software program SPSS was used to analyze all data (IBM SPSS version 28).

## 3 RESULTS

Descriptive characteristics and baseline spirometry are summarized in Table 1. In asthmatic subjects, FEV_1_/FVC and FEF_25‐75%_ were lower than predicted values (*p* < 0.05). Significant BD_res_ was defined as >12% increase in FEV_1_ or FVC after bronchodilator inhalation (Standardization of Spirometry, 1995). Six out of the ten asthmatic subjects demonstrated BD_res_ (range, +0.9% to +30% increase in FEV_1_). However, FEV_1_ decreased by at least 15% from baseline after the incremental exercise test (range, −15% to −60%) in all asthmatic subjects. Altogether, whereas airway function was normal in the control group, asthmatic subjects demonstrated mildly reduced baseline airway function and mild‐to‐moderate airways hyperresponsiveness. Maximal oxygen uptake and power output (watts·kg^−1^) were not different between control and asthmatic subjects.

**TABLE 1:** Descriptive characteristics for control and asthmatic subjects.

	Control group (*n* = 9)	Asthmatic group (*n* = 10)
Male/female	4/5	7/3
Age, years	25.4 ± 7.8	26.5 ± 8.0
Height, m	1.72 ± 0.11	1.72 ± 0.07
Weight, kg	65.3 ± 11.3	80.8 ± 16.7*
BMI	21.9 ± 2.0	27.1 ± 4.2*
FVC, l	4.76 ± 1.08 (102.0 ± 12.7)	5.02 ± 0.69 (108.1 ± 19.7)
FEV_1_, l	3.79 ± 0.77 (96.5 ± 12.2)	3.65 ± 0.69 (92.5 ± 17.2)
FEV_1_/FVC	0.80 ± 0.04 (93.8 ± 4.8)	0.73 ± 0.09*, ^†^ (85.5 ± 10.5)*
FEF_25‐75%_, L·s^−1^	3.51 ± 0.69 (82.1 ± 16.7)	2.95 ± 1.17^†^ (67.2 ± 24.0)
PEF, L·s^−1^	7.96 ± 2.12 (93.4 ± 15.3)	8.41 ± 1.62 (93.0 ± 11.0)
$\overset{\cdot }{V}O_{2}\text{peak}$, mL·kg^−1^·min^−1^	41.1 ± 8.3 (107.7 ± 17.8%)	42.7 ± 9.6 (103.7 ± 21.3%)
Peak power output, watts·kg^−1^	3.04 ± 0.64	3.04 ± 0.81
BD reversibility, % change FEV_1.0_	+4.29 ± 3.57	+13.2 ± 8.9*
EIB, % change FEV_1.0_	−9.84 ± 4.90	−28.0 ± 13.5*

*Note*: Values in parentheses are percent predicted. Data are means ± SD.

Abbreviations: BD, bronchodilator; BMI, body mass index; EIB, exercise‐induced bronchoconstriction; FEF_25‐75%_, forced expiratory flow between 25% and 75% FVC; FEV_1_, forced expiratory volume 1 s; FVC, forced vital capacity; PEF, peak expiratory flow; $\overset{\cdot }{V}O_{2}\text{peak}$, maximal oxygen uptake.

* *p* < 0.05 versus control group.

^†^ *p* < 0.05 versus predicted.

### 3.1 Pulmonary function

**FIGURE 2:**
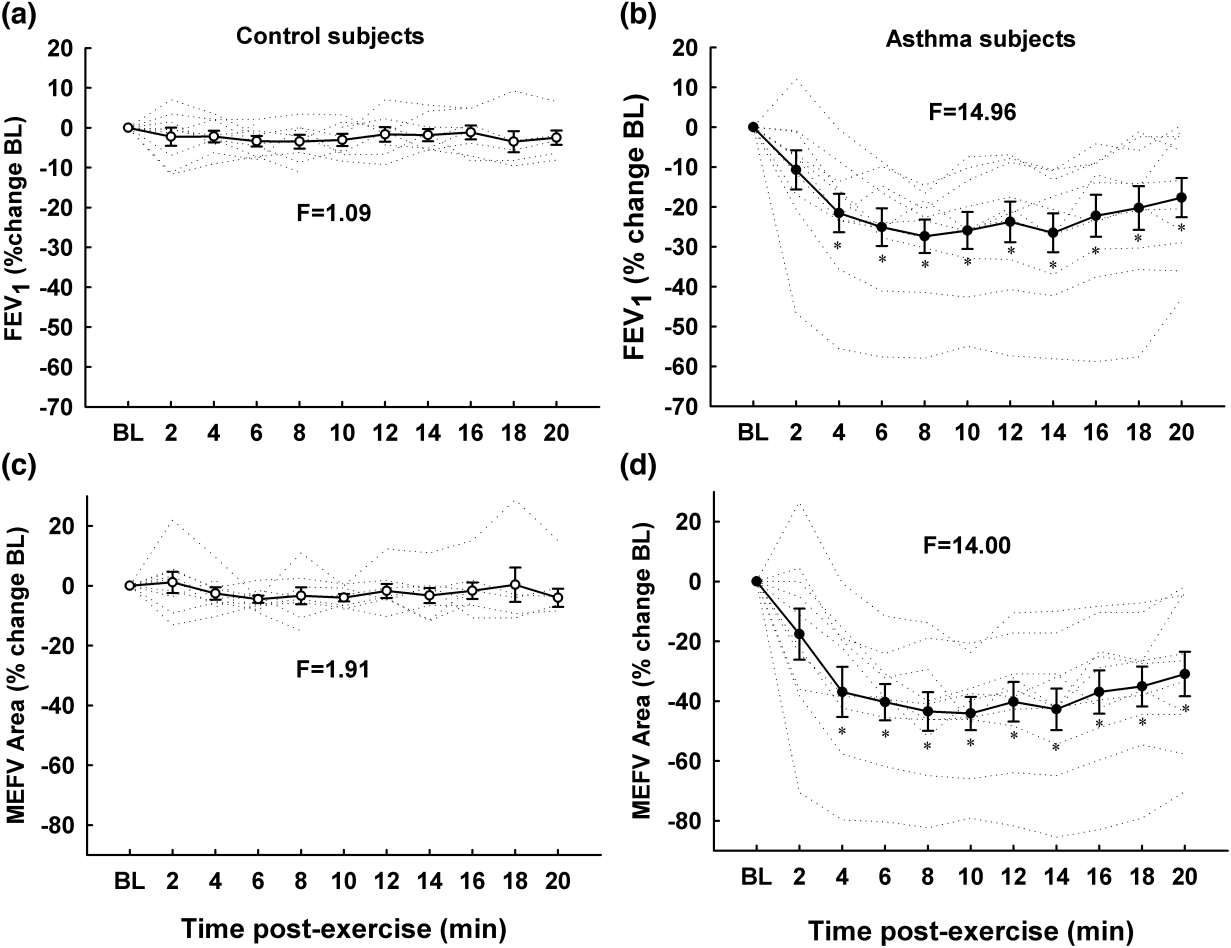
Forced expiratory volume in 1.0 second (FEV_1_) and area of maximal expiratory flow‐volume curves (MEFV) in control (a, c) and asthmatic (b, d) subjects after a six‐minute exercise challenge on a cycle ergometer. FEV_1_ and area of the MEFV curve decreased significantly after exercise in asthmatic subjects. Data are presented as mean ± SD. *p < 0.05 versus baseline.

Figure 2a–d shows individual subject and group mean values for FEV_1_ and area under the MEFV curve expressed as percentage changes from baseline in control and asthmatic subjects. In control subjects, post‐exercise FEV_1_ and MEFV area were not different from baseline (Panel A & C). In asthmatic subjects, post‐exercise FEV_1_ was decreased from baseline at all timepoints (*F* = 14.96; *p* < 0.001). On average, nadir FEV_1_ occurred 11.2 ± 4.7 min after exercise (range, 4–20 min post‐exercise). Group mean post‐exercise nadir FEV_1_ was decreased by 29.9 ± 13.2% (range, −14.6% to −58.8%) from baseline (3.48 ± 0.74 to 2.24 ± 0.59 [SD] L for baseline and post‐exercise nadir, respectively, *P* < 0.001). FEV_1_ was strongly associated with area under the MEFV curve (*r* ^2^ = 0.94; *p* < 0.001). Additional spirometric results are shown in Table 2. FEV_1_, FEV_1_/FVC, and PEF were significantly decreased after exercise in asthmatic subjects whereas they were unchanged by exercise in control subjects.

**TABLE 2:** Pulmonary function at baseline and after exercise in control and asthmatic subjects.

Variable	Time post‐exercise										
	Baseline	2 min	4 min	6 min	8 min	10 min	12 min	14 min	16 min	18 min	20 min
FEV_1_, l											
Control	3.84 ± 0.9	3.74 ± 0.9	3.74 ± 0.8	3.70 ± 0.9	3.71 ± 0.8	3.70 ± 0.8	3.74 ± 0.8	3.75 ± 0.8	3.76 ± 0.8	3.71 ± 0.8	3.71 ± 0.8
Asthma	3.48 ± 0.7	3.11 ± 0.9	2.67 ± 0.8*	2.59 ± 0.7*	2.52 ± 0.7*	2.56 ± 0.7*	2.63 ± 0.7*	2.49 ± 0.7*	2.68 ± 0.8*	2.75 ± 0.8*	2.84 ± 0.7*
FVC, l											
Control	5.00 ± 1.4	4.83 ± 1.4	4.84 ± 1.3	4.78 ± 1.3*	4.86 ± 1.4	4.80 ± 1.3*	4.84 ± 1.3	4.87 ± 1.3	4.85 ± 1.3	4.89 ± 1.3	4.83 ± 1.2
Asthma	5.14 ± 0.7	4.74 ± 0.8	4.37 ± 0.9	4.35 ± 0.9	4.26 ± 0.9	4.29 ± 0.9	4.38 ± 0.9	4.35 ± 0.9	4.48 ± 0.9	4.55 ± 0.8	4.67 ± 0.8
FEV_1_/FVC											
Control	0.78 ± 0.08	0.79 ± 0.08	0.79 ± 0.08	0.79 ± 0.08	0.78 ± 0.09	0.78 ± 0.07	0.78 ± 0.08	0.78 ± 0.08	0.79 ± 0.06	0.79 ± 0.05	0.79 ± 0.06
Asthma	0.67 ± 0.09	0.65 ± 0.09	0.60 ± 0.08*	0.59 ± 0.06*	0.59 ± 0.07*	0.59 ± 0.07*	0.59 ± 0.06*	0.57 ± 0.07*	0.59 ± 0.08*	0.59 ± 0.09*	0.60 ± 0.07*
PEF, L·s^−1^											
Control	9.13 ± 2.5	8.54 ± 2.4	8.64 ± 2.5	8.47 ± 2.4	8.46 ± 2.3	8.44 ± 2.3	8.51 ± 2.1	8.45 ± 2.3	8.41 ± 1.8	8.27 ± 2.2	8.60 ± 2.1
Asthma	7.86 ± 2.2	7.14 ± 2.4	6.35 ± 2.3*	5.86 ± 2.0*	6.00 ± 2.3*	5.60 ± 1.9*	6.60 ± 2.2*	5.80 ± 2.0*	5.94 ± 2.1*	5.82 ± 2.1*	6.18 ± 1.8*

*Note*: Data are means ± SD.

* *p* < 0.05 versus baseline.

**FIGURE 3:**
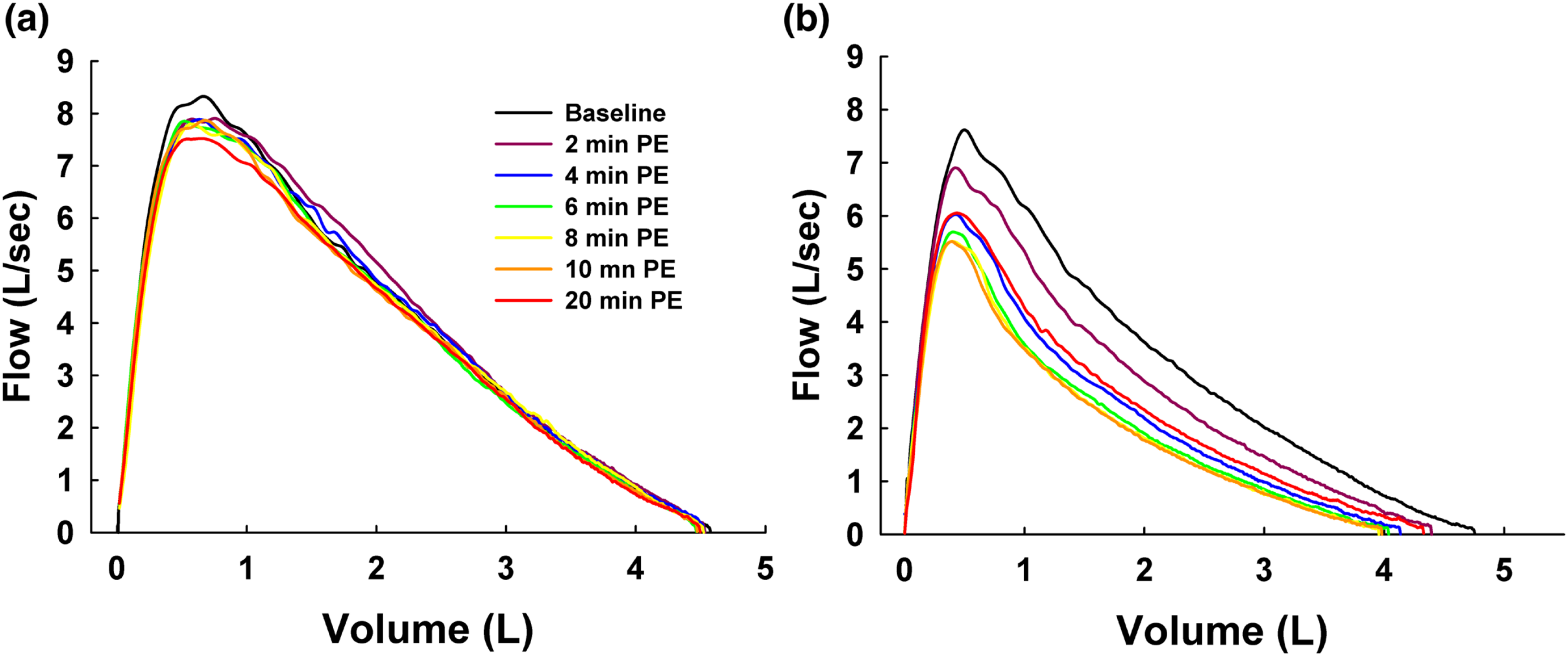
Ensemble‐averaged maximal expiratory flow‐volume (MEFV) curves in (a) control and (b) asthmatic subjects at baseline and at multiple timepoints after exercise (post‐exercise [PE]). In asthmatic subjects, the post‐exercise MEFV curves were substantially smaller than the baseline MEFV curve.

Figure 3a,b shows ensemble‐averaged MEFV curves at baseline and 2, 4, 6, 8, 10, and 20 min after exercise in control and asthma subjects. In control subjects, the post‐exercise MEFV curves were similar to the baseline curve, in support of the unchanged spirometry summarized in Table 2. In asthmatic subjects, the MEFV curves depict a progressive airway narrowing up to 10 min post exercise. The MEFV curve from 20 min post‐exercise shows that the airways had dilated compared with their maximal narrowing (at 10 min) but still remained smaller than the baseline MEFV curve.

### 3.2 Slope‐ratio

**FIGURE 4:**
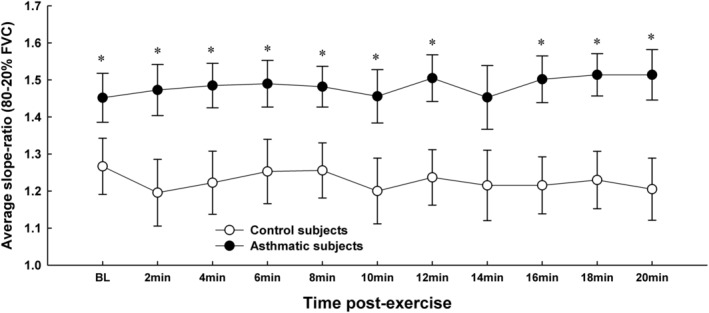
Average slope‐ratio between 80% and 20% of forced vital capacity at baseline and at multiple timepoints after exercise in control and asthmatic subjects. Slope‐ratio was significantly larger in the asthmatic subjects than the control subjects at all timepoints. Data are presented as mean ± SD. *p < 0.05 versus control group.

Figure 4 depicts the average SR between 80% and 20% FVC at baseline and at all post‐exercise timepoints in control and asthmatic subjects. There was a significant main effect of group (*F* = 10.22; *p* = 0.005). On average, SR was 21 ± 2.3% larger in asthmatic subjects than control subjects (1.48 ± 0.02 vs. 1.23 ± 0.02 [SD] for asthma vs. control; *p* < 0.05 at 10 out of the 11 timepoints). However, in both subject groups, post‐exercise SR was not different from baseline at any time point after exercise.

**FIGURE 5:**
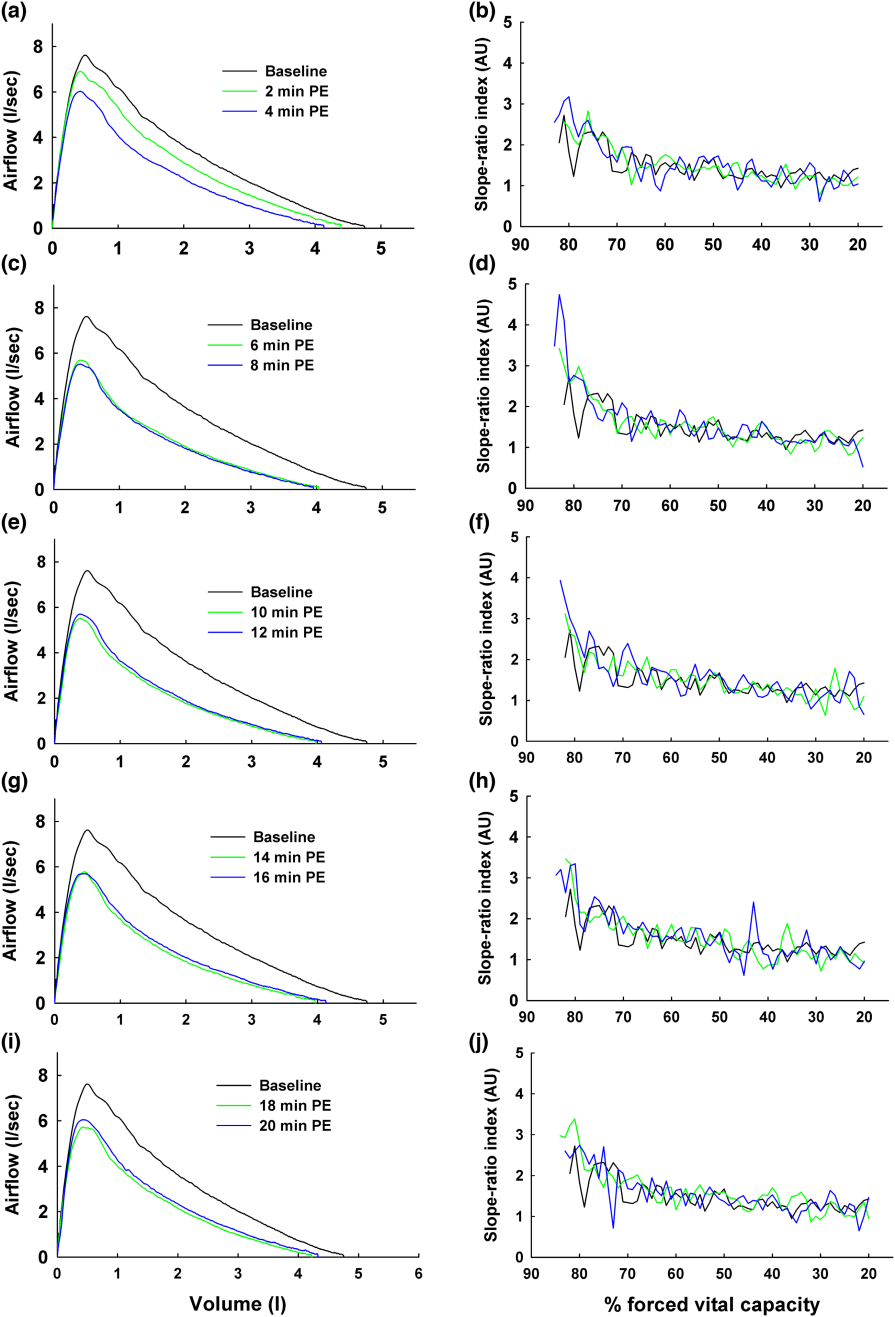
Baseline and post‐exercise maximal expiratory flow‐volume curves (a, c, e, g, i) and slope‐ratio index plotted in 1% volume increments between peak expiratory flow and 20% of vital capacity (b, d, f, h, j) in asthmatic subjects. Each panel depicts responses from baseline and at two post‐exercise timepoints. Note that post‐exercise slope‐ratio at high lung volumes was larger than baseline at all post‐exercise timepoints.

Figure 5a–j presents ensemble‐averaged MEFV curves and group mean SR in continuous, 1% volume increments at baseline and at each post‐exercise timepoint in the asthmatic subjects. Each panel depicts the results at baseline and at two post‐exercise timepoints. The most notable finding shown in these figures is that post‐exercise SR at high lung volumes (above approximately 75% of FVC) was increased from baseline at all post‐exercise timepoints. However, post‐exercise SR at lung volumes below ~75% FVC were not different from baseline.

**FIGURE 6:**
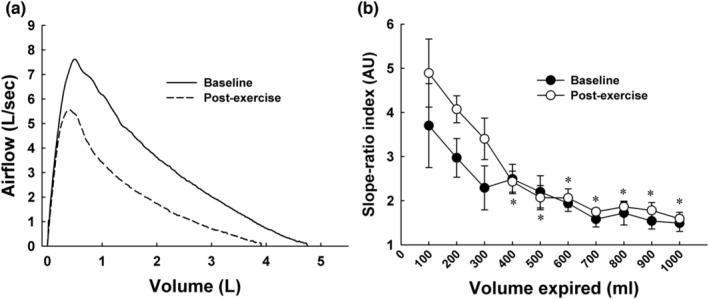
(a) Ensemble‐averaged maximal expiratory flow‐volume (MEFV) curve at baseline and at the time of maximal post‐exercise airway narrowing in subjects with asthma. (b) Asthmatic group mean slope‐ratio index in 100 mL volume increments beginning at peak expiratory flow and ending after 1000 mL expired. Slope‐ratio in the first 300 mL of expired volume following peak expiratory flow was increased after exercise. (a) *p* < 0.05 versus 100 mL; (b) *p* < 0.05 versus 200 mL; (c) *p* < 0.05 versus 300 mL; (d) *p* < 0.05 versus 400 mL.

Since FVC decreased after exercise in the subjects with asthma, absolute post‐exercise lung volumes were larger than the baseline volumes at any given percent of FVC (e.g., 80% of 5.0 L = 1 L expired; 80% of 4.0 L = 0.8 L expired). We thus compared baseline SR with post‐exercise SR at the same absolute lung volumes. Figure 6a shows the ensemble‐averaged MEFV curve at baseline and from the post‐exercise MEFV curve at the time of maximal airway narrowing (i.e., lowest post‐exercise FEV_1_). In Figure 6b, group mean SR is shown in 100 mL volume increments beginning at PEF and ending 1 liter below PEF. Repeated measures ANOVA revealed a significant main effect for lung volume (*F* = 8.56; *p* < 0.001) whereas the main effect for time (baseline vs. post‐exercise) was not significant (*p* = 0.073). Despite the lack of a significant effect of time, mean post‐exercise SR was increased by 38% from baseline at the three highest lung volumes (100, 200, 300 mL) (SR = 3.0 ± 2.1 vs. 4.1 ± 1.8 [SD] for baseline vs. post‐exercise). At lung volumes lower than 300 mL, baseline and post‐exercise SR were nearly identical. We also correlated the maximum absolute change in FEV_1_ after exercise (nadir post‐exercise FEV_1_ – baseline FEV_1_) with the change in SR measured at the three highest lung volumes. The change in FEV_1_ was not associated with the change in SR at any of the lung volumes (∆ FEV_1_ vs. ∆ SR at 100 mL, *R* ^2^ = 0.071, *p* = 0.456; ∆ FEV_1_ vs. ∆ SR at 200 mL, *R* ^2^ = 0.069, *p* = 0.464; ∆ FEV_1_ vs. ∆ SR at 300 mL, R^2^ = 0.0–0.274, *p* = 0.120).

### 3.3 Exercise responses

Ventilatory and metabolic responses before and during exercise are shown in Table 3. The exercise data are reported as the average values obtained during the final 3 min of exercise. There were no differences in exercise ${\dot{V}}_{E}$, ${\dot{V}}_{O2}$, ${\dot{V}}_{\mathrm{CO}2}$, ${\dot{V}}_{E}/{\dot{V}}_{\mathrm{CO}2}$, respiratory exchange ratio, or exercise intensity expressed as percent of V̇_O2peak_ or peak power output between control and asthmatic subjects.

**TABLE 3:** Ventilatory and metabolic variables at baseline and during exercise in control and asthmatic subjects.

	Control	Asthma
${\overset{\cdot }{V}}_{E}$, L·min^−1^		
Baseline	14.0 ± 2.8	15.5 ± 6.5
Exercise	80.9 ± 16.6	95.9 ± 14.4
${\overset{\cdot }{V}}_{E}$/kg		
Baseline	0.21 ± 0.02	0.19 ± 0.06
Exercise	1.25 ± 0.23	1.23 ± 0.32
${\overset{\cdot }{V}}_{E}$/MVV		
Baseline	0.09 ± 0.02	0.11 ± 0.04
Exercise	0.54 ± 0.10	0.69 ± 0.18*
VT, L·breath^−1^		
Baseline	1.0 ± 0.5	1.4 ± 0.7
Exercise	2.5 ± 0.8	2.8 ± 0.6
Fb, breaths·min^−1^		
Baseline	15.6 ± 4.3	12.0 ± 3.4
Exercise	34.9 ± 8.1	35.3 ± 8.8
${\overset{\cdot }{V}}_{O2}$, mL·kg^−1^·min^−1^		
Baseline	6.5 ± 0.6	5.4 ± 1.3*
Exercise	35.8 ± 7.0	36.7 ± 9.3
${\overset{\cdot }{V}}_{\mathrm{CO}2}$, mL·kg^−1^·min^−1^		
Baseline	5.4 ± 0.8	4.9 ± 1.5
Exercise	39.9 ± 9.2	40.6 ± 11.4
${\overset{\cdot }{V}}_{E}/{\overset{\cdot }{V}}_{\mathrm{CO}2}$		
Baseline	40.7 ± 6.7	38.3 ± 5.4
Exercise	32.0 ± 5.1	30.8 ± 3.9
RER		
Baseline	0.84 ± 0.16	0.91 ± 0.16
Exercise	1.11 ± 0.08	1.10 ± 0.07
% PPO	85.4 ± 1.7	85.8 ± 2.2
% $\overset{\cdot }{V}O_{2}\text{peak}$	85.6 ± 13.1	85.8 ± 8.3

*Note*: Data are means ± SD.

Abbreviations: MVV, maximal voluntary ventilation; PPO, peak power output; RER, respiratory exchange ratio; ${\overset{\cdot }{V}}_{\mathrm{CO}2}$, carbon dioxide production; ${\overset{\cdot }{V}}_{E}$, minute ventilation; ${\overset{\cdot }{V}}_{O2}$, oxygen consumption; VT, tidal volume; fb, breathing frequency.

* *p* < 0.05 versus Control.

## 4 DISCUSSION

We calculated the SR‐index at baseline and serially after exercise in a group of asthmatic adults with EIB and in a control group of non‐asthmatics. Our findings provide new evidence suggesting that the SR‐index might be sufficiently sensitive to detect increased airway caliber heterogeneity during an episode of EIB. However, the post‐exercise increase in SR was only evident at high lung volumes, indicating a steeper slope for airflow versus volume during the transition from the effort‐dependent to ‐independent lung volume region. Our findings also support previous work that overall SR is increased in persons with asthma. Finally, in the subjects with asthma, SR exhibited a clear volume dependence, decreasing progressively as lung volume decreased. We conclude that EIB alters airway mechanical properties such that the overall increases in resistance are accompanied by increased airway caliber heterogeneity that can be identified using the SR analysis.

Our principal new finding is that the MEFV curve becomes more curvilinear at high lung volumes during an episode of EIB (Figures 5 and 6). MEFV curve configuration at lung volumes below ~75% of FVC was not affected by the exercise‐induced airway narrowing. To our knowledge, the only previous analysis of the SR‐index after exercise in asthma is reported in Mead's original description of the method (Mead, 1978). In two out of the four asthmatic subjects in Mead's analysis, SR was clearly increased from control conditions at high lung volumes (70%–80% of vital capacity). O'Donnel and colleagues reported SR‐volume curves in five control and five asthmatic subjects at baseline and after histamine inhalation (O'Donnel et al., 1986). Following histamine, SR at high lung volumes increased in two out of the five asthmatic subjects. Dominelli et al. reported an unchanged SR after a eucapnic voluntary hyperpnea (EVH) challenge in a group of adult asthmatics (Dominelli et al., 2016). A limitation of these previous studies is that SR was reported only once after each intervention. In contrast, our subjects performed serial maximal forced expirations every 2 min after exercise, allowing for a longitudinal analysis of post‐exercise SR. Overall, SR was quite stable during the 20‐min recovery period in our subjects. The stable post‐exercise SR contrasts with the progressive airway narrowing during the first 10–15 min after exercise, followed by a gradual dilation during the remaining recovery time. These findings suggest that the increased airway caliber inhomogeneity preceded the airway narrowing and that recovery of the inhomogeneity lagged recovery of the narrowing.

Given that the same physiological sequence of events is responsible for the airway narrowing after both exercise and EVH (Hallstrand et al., 2018), the lack of concordance between our findings and those of Dominelli (Dominelli et al., 2016) is intriguing. We suggest several possible explanations for the different findings. From a methodological standpoint, we calculated SR beginning at PEF whereas it was calculated beginning at 80% of FVC in the previous study. Also, in the previous study, subjects performed a single maximal forced exhalation at approximately 10 min after the EVH challenge. It is possible that the single post‐EVH MEFV curve did not capture a change in SR. Physiologically, there are several important exercise responses, not present during EVH, that might bring about unequal airway narrowing. Certainly, the increases in cardiac output and pulmonary vascular pressures during high‐intensity exercise do not occur during EVH. As fluid flux from the pulmonary vessels is dictated by classic Starling forces (Staub, 1974), increased pulmonary vascular pressures with exercise surely increase net outward fluid flux. On the one hand, the exercise workloads and duration, and cardiac outputs in our subjects were less than those required for significant extravascular fluid accumulation (Bates et al., 2011). On the other hand, if the increased fluid extravasation is not balanced by an equal increase in clearance by the lymph system, the added fluid will begin accumulating around the airways and larger pulmonary vessels (so‐called “cuffing”) (Staub et al., 1967). Although speculative, such cuffing might increase airway caliber inhomogeneity enough to widen the distribution of time constants during forced expiration. Increased sympathetic nervous system activity and the rise in circulating epinephrine during exercise—but not during EVH—might contribute to airway caliber heterogeneity if perfusion to the airways is unequal or the airways are differentially dilated by the epinephrine.

In our asthmatic subjects, SR decreased progressively during the forced exhalation both at baseline and after exercise (Figures 5 and 6). No such volume dependence was seen in control subjects. An earlier study also showed that the SR‐volume relationship was positive in asthmatics whereas it was nearly flat in non‐asthmatics (Dominelli et al., 2016). In other work, histamine inhalation altered the configuration of the MEFV curve in five asthmatics by causing a clear SR‐volume dependence that was not present at baseline (O'Donnel et al., 1986). In addition, MEFV curve concavity decreased following acute inhalation of bronchodilator in asthmatic children (Patel et al., 2008) and adults (Ohwada & Takahashi, 2012) and after inhaled corticosteroid treatment in adult asthmatics (Kraan et al., 1989). Collectively, there is compelling evidence that asthma causes increases in both MEFV curvature and volume dependence of the curvature. Our current findings show that these phenotypic effects on the MEFV curve are further magnified during an episode of EIB.

What do the present findings suggest regarding the effect of EIB on the structural and functional determinants of expired airflow? First, the degree of curvature might reflect the overall extent of airway narrowing (Spycher et al., 2012). However, our finding of a generally stable post‐exercise SR throughout the recovery period does not support this notion (Figure 5). Second, the higher overall values for SR in the subjects with asthma (Figure 4) and the increased SR during early expiration after exercise suggest more heterogeneous airway emptying during the forced expiration. There is a sound theoretical argument that airflow during a maximum forced expiration becomes curvilinear as lung unit emptying becomes more heterogeneous. In particular, Mead's detailed analysis predicts that non‐homogenous rates of emptying among lung regions will preferentially increase SR at high lung volumes (Mead, 1978). As well, early airway closure is predicted to increase SR at high lung volumes (Pride & Macklem, 1985). Indeed, in our subjects, the substantial decreases in FVC after exercise is compatible with this prediction (Figure 3b). Many previous studies have reported decreases in FVC after exercise or EVH in adults with asthma (Haverkamp et al., 2005; Haverkamp et al., 2021). The reduced FVC is likely due to peripheral airway inflammation and narrowing, which causes early airway closure and an accompanying increase in residual volume. We did not measure lung volumes in this study; however, future studies that couple MEFV curve SR with lung volumes would be insightful. In addition to the physiologically‐based arguments, mathematical modeling predicts that heterogeneous airway resistances cause MEFV curves to become more curvilinear (Lambert, 1990). Finally, imaging studies using hyperpolarized helium and the multiple inert gas elimination technique demonstrate that airway narrowing causes heterogeneous ventilation distribution in subjects with asthma (Muñoz et al., 2008; Samee et al., 2003). Collectively, several types of evidence have shown that the airway narrowing in asthma is heterogeneous. Our current findings provide evidence that the SR analysis applied to MEFV curves can detect acute increases in airway caliber heterogeneity.

There are several causes of heterogeneous airway caliber in the asthmatic. Heterogeneities in (1) airway geometry and thickness (Moreno et al., 1986), (2) luminal fluids (Yager et al., 1989), (3) regional differences in outward tethering forces (Venegas et al., 2005), and (4) variable airway smooth muscle shortening (King et al., 2004; Lutchen et al., 2001) all contribute to the stochastic nature of airway narrowing (Bates, 1993). Actually, given the complexity of, and interaction among, these determinants of airway caliber, regional heterogeneity in airway resistances is neither surprising nor unexpected. For these reasons, we think it is reasonable to suggest that the increased MEFV curvature was primarily due to unequal airway narrowing and the resultant increase in heterogeneous airway emptying.

### 4.1 Relevance of SR‐index

The SR‐index provides a quantitative, unbiased, metric of the shape of the MEFV curve. Such a quantitative “picture” of the MEFV curve could aid in the diagnostic and prognostic management of respiratory disease. Certainly, respiratory therapists and pulmonologists make a qualitative assessment of MEFV curve conformation. This subjective assessment is assimilated with the objective measures to inform final interpretation. Furthermore, in the early stages of chronic obstructive pulmonary disease when FEV_1_ is within normal range, MEFV curve concavity might be a more sensitive index of nascent airway obstruction than traditional spirometric measures (Johns et al., 2017). Other findings suggest that MEFV curve phenotype might also be a more sensitive test for identifying early pathophysiological changes in respiratory system function in populations such as asymptomatic smokers and differentiating pathophysiological from normal effects of aging on airway function (Dominelli et al., 2015). Unlike other methods for quantitating MEFV curvilinearity, the SR‐index provides insight into airway and lung physiology throughout the vital capacity.

### 4.2 Caveats and limitations

Gas compression during the maximal forced expirations was not accounted for in this study. Thus, the MEFV curves are likely more concave than they would have been if gas compression had been quantified (Ingram & Schilder, 1966). We appreciate that airflow during the first ~20% of the forced expiration is effort‐dependent. While acknowledging our assumption that maximum effort was achieved during the maximal forced expirations, we think that analyzing this portion of the MEFV curve provides additional insight into airway mechanical function beyond the effort‐independent lung volumes. Also, since submaximal expiratory effort would cause reduced MEFV curve slope at high lung volumes (vs. increased slope), it would also lead to underestimated (vs. overestimated) values for SR. Finally, in the absolute volume‐based analysis of the SR (Figure 6), we assumed that total lung capacity was unchanged after exercise. There are no reports of decreases in TLC accompanying an episode of EIB. However, two previous publications reported an increased TLC in asthmatic subjects after an exercise bout (Anderson et al., 1972; Freedman et al., 1975). In both publications, the EIB was more severe than seen in our subjects and the increased TLC was not evident in all subjects. As well, in patients with asthma, plethysmography is prone to overestimating lung volumes due to incomplete transmission of alveolar pressure to mouth pressure (Pride & Macklem, 1985).

## 5 CONCLUSIONS

We analyzed SR‐volume curves before and serially after high‐intensity exercise in non‐asthmatic and asthmatic adults. In our asthmatic subjects, SR at high lung volumes increased after exercise. The increased SR supports findings from both physiological and imaging studies showing that the extent of airway narrowing in EIB is heterogeneous in adults with asthma (James et al., 2022; Samee et al., 2003). Thus, the SR analysis appears to provide a simple, inexpensive technique for characterizing the effects of acute bronchoconstriction on ventilation heterogeneity in patients with pulmonary disease.

## AUTHOR CONTRIBUTIONS

Hans Christian Haverkamp conceived of manuscript idea and supervised the project. Oksana Klimenko and Peter Luu developed the slope‐ratio analysis and organized data. Hans Christian Haverkamp, Oksana Klimenko, Peter Luu, Nathan Noggle, Gregory Petrics analyzed data. Hans Christian Haverkamp and Paolo Dominelli prepared manuscript. All authors provided feedback and helped shape the research, analyses, and manuscript.

## ETHICS STATEMENT

This study and all procedures were approved by the Institutional Review Boards for research involving human subjects, and it was conducted in compliance with the Declaration of Helsinki.

## FUNDING INFORMATION

Research reported in this publication was supported by an Institutional Development Award (IDeA) from the National Institute of General Medical Sciences of the National Institutes of Health under grant number P20GM103449. Its contents are solely the responsibility of the authors and do not necessarily represent the official views of NIGMS or NIH.

## References

[phy215614-bib-0001] Anderson, S. A. , McEvoy, J. D. S. , & Bianco, S. (1972). Changes in lung volumes and airway resistance after exercise in asthmatic subjects. The American Review of Respiratory Disease, 106, 30–37. 4624795 10.1164/arrd.1972.106.1.30

[phy215614-bib-0002] Bates, J. H. T. (1993). Stochastic model of the pulmonary airway tree and its implications for bronchial responsiveness. Journal of Applied Physiology, 75(6), 2493–2499. 8125867 10.1152/jappl.1993.75.6.2493

[phy215614-bib-0003] Bates, M. L. , Farrell, E. T. , & Eldridge, M. W. (2011). The curious question of exercise‐induced pulmonary edema. Pulmonary Medicine, 361931. 10.1155/2011/361931 21660232 PMC3109354

[phy215614-bib-0004] Bhatt, S. P. , Bodduluri, S. , Raghav, V. , Bhakta, N. R. , Wilson, C. G. , Kim, Y. I. , Eberlein, M. , Sciurba, F. C. , Han, M. K. , Dransfield, M. T. , & Nakhmani, A. (2019). The peak index: Spirometry metric for airflow obstruction severity and heterogeneity. Annals of the American Thoracic Society, 16(8), 982–989. 30865842 10.1513/AnnalsATS.201811-812OC PMC6774744

[phy215614-bib-0005] Crimi, E. , Pellegrino, R. , Smeraldi, A. , & Brusasco, V. (2002). Exercise‐induced bronchodilation in natural and induced asthma: Effects on ventilatory response and performance. Journal of Applied Physiology, 92(6), 2353–2360. 12015347 10.1152/japplphysiol.01248.2001

[phy215614-bib-0006] Dominelli, P. B. , Foster, G. E. , Guenette, J. A. , Haverkamp, H. C. , Eves, N. D. , Dominelli, G. S. , Henderson, W. R. , O'Donnell, D. E. , & Sheel, A. W. (2015). Quantifying the shape of the maximal expiratory flow‐volume curve in mild COPD. Respiratory Physiology & Neurobiology, 219, 30–35. 26275685 10.1016/j.resp.2015.08.002

[phy215614-bib-0007] Dominelli, P. B. , Molgat‐Seon, Y. , Foster, G. E. , Dominelli, G. S. , Haverkamp, H. C. , Henderson, W. R. , & Sheel, A. W. (2016). Quantifying the shape of maximal expiratory flow‐volume curves in healthy humans and asthmatic patients. Respiratory Physiology & Neurobiology, 220, 46–53. 26388199 10.1016/j.resp.2015.09.007 PMC6238952

[phy215614-bib-0008] Freedman, S. , Tattersfield, A. E. , & Pride, N. B. (1975). Changes in lung mechanics during asthma induced by exercise. Journal of Applied Physiology, 38(6), 974–982. 1141137 10.1152/jappl.1975.38.6.974

[phy215614-bib-0009] Hallstrand, T. S. , Leuppi, J. D. , Joos, G. , Hall, G. L. , Carlsen, K. H. , Kaminsky, D. A. , Coates, A. L. , Cockcroft, D. W. , Culver, B. H. , Diamant, Z. , Gauvreau, G. M. , Horvath, I. , de FHC, J. , Laube, B. L. , Sterk, P. J. , & Wanger, J. (2018). ERS technical standard on bronchial challenge testing: Pathophysiology and methodology of indirect airway challenge testing. The European Respiratory Journal, 52, 1801033. 30361249 10.1183/13993003.01033-2018

[phy215614-bib-0010] Hallstrand, T. S. , Moody, M. W. , Wurfel, M. M. , Schwartz, L. B. , Henderson, W. R. , & Aitken, M. L. (2005). Inflammatory basis of exercise‐induced bronchoconstriction. American Journal of Respiratory and Critical Care Medicine, 172(6), 679–686. 15947280 10.1164/rccm.200412-1667OC PMC2041799

[phy215614-bib-0011] Haverkamp, H. C. , Dempsey, J. A. , Miller, J. D. , Romer, L. M. , Pegelow, D. F. , Lovering, A. T. , & Eldridge, M. W. (2005). Repeat exercise normalizes the gas‐exchange impairment induced by a previous exercise bout in asthmatic subjects. Journal of Applied Physiology, 99(5), 1843–1852. 16037395 10.1152/japplphysiol.01399.2004

[phy215614-bib-0012] Haverkamp, H. C. , Kaminsky, D. A. , McPherson, S. M. , & Irvin, C. G. (2021). Spirometric response to bronchodilator and eucapnic voluntary hyperpnea in adults with asthma. Respiratory Care, 66(8), 1282–1290. 34006592 10.4187/respcare.08421 PMC9994366

[phy215614-bib-0013] Hoesterey, D. , Das, N. , Janssens, W. , Buhr, R. G. , Martinez, F. J. , Cooper, C. B. , Tashkin, D. P. , & Barjaktarevic, I. (2019). Spirometric indices of early airflow impairment in individuals at risk of developing COPD: Spirometry beyond FEV1/FVC. Respiratory Medicine, 156, 58–68. 31437649 10.1016/j.rmed.2019.08.004 PMC6768077

[phy215614-bib-0014] Ingram, R. H. , & Schilder, D. P. (1966). Effect of gas compression on pulmonary pressure, flow, and volume relationship. Journal of Applied Physiology, 21(6), 1821–1826. 5929308 10.1152/jappl.1966.21.6.1821

[phy215614-bib-0015] James, A. L. , Donovan, G. M. , Green, F. H. Y. , Mauad, T. , Abramson, M. J. , Cairncross, A. , Noble, P. B. , & Elliot, J. G. (2023). Heterogeneity of airway smooth muscle remodelling in asthma. American Journal of Respiratory and Critical Care Medicine, 207(4), 452–460. 10.1164/rccm.202111-2634OC 36399661

[phy215614-bib-0016] Johns, D. P. , Das, A. , Toelle, B. G. , Abramson, M. J. , Marks, G. B. , Wood‐Baker, R. , & Walters, E. H. (2017). Improved spirometric detection of small airway narrowing: Concavity in the expiratory flow–volume curve in people aged over 40 years. International Journal of Chronic Obstructive Pulmonary Disease, 12, 3567–3577. 29263661 10.2147/COPD.S150280 PMC5732561

[phy215614-bib-0017] Johns, D. P. , Walters, J. A. E. , & Haydn, W. E. (2014). Diagnosis and early detection of COPD using spirometry. Journal of Thoracic Disease, 6, 1557–1569. 25478197 10.3978/j.issn.2072-1439.2014.08.18 PMC4255165

[phy215614-bib-0018] King, G. G. , Carroll, J. D. , Müller, N. L. , Whittall, K. P. , Gao, M. , Nakano, Y. , & Paré, P. D. (2004). Heterogeneity of narrowing in normal and asthmatic airways measured by HRCT. The European Respiratory Journal, 24(2), 211–218. 15332387 10.1183/09031936.04.00047503

[phy215614-bib-0019] Klansky, A. , Irvin, C. , Morrison‐Taylor, A. , Ahlstrand, S. , Labrie, D. , & Haverkamp, H. C. (2016). No effect of elevated operating lung volumes on airway function during variable workrate exercise in asthmatic humans. Journal of Applied Physiology, 121(1), 89–100. 27150833 10.1152/japplphysiol.00538.2015 PMC4967247

[phy215614-bib-0020] Kraan, J. , van der Mark, T. W. , & Koeter, G. H. (1989). Changes in maximum expiratory flow‐volume curve configuration after treatment with inhaled corticosteroids. Thorax, 44(12), 1015–1021. 2533411 10.1136/thx.44.12.1015 PMC1020877

[phy215614-bib-0021] Lambert, R. K. (1990). Simulation of the effects of mechanical nonhomogeneities on expiratory flow from human lungs. Journal of Applied Physiology, 68(6), 2550–2563. 2200781 10.1152/jappl.1990.68.6.2550

[phy215614-bib-0022] Lutchen, K. R. , Kaczka, D. W. , Israel, E. , Suki, B. , & Ingenito, E. P. (2001). Airway constriction pattern is a central component of asthma severity: The role of deep inspirations. American Journal of Respiratory and Critical Care Medicine, 164, 2017–2215. 10.1164/ajrccm.164.2.2008119 11463589

[phy215614-bib-0023] Mead, J. (1978). Analysis of the configuration of maximum expiratory flow‐volume curves. Journal of Applied Physiology: Respiratory, Environmental and Exercise Physiology, 44(2), 156–165. 632154 10.1152/jappl.1978.44.2.156

[phy215614-bib-0024] Milanese, M. , Saporiti, R. , Bartolini, S. , Pellegrino, R. , Baroffio, M. , Brusasco, V. , & Crimi, E. (2009). Bronchodilator effects of exercise hyperpnea and albuterol in mild‐to‐moderate asthma. Journal of Applied Physiology, 107(2), 494–499. 19541736 10.1152/japplphysiol.00302.2009

[phy215614-bib-0025] Miller, M. R. , Hankinson, J. , Brusasco, V. , Burgos, F. , Casaburi, R. , Coates, A. , Crapo, R. , Enright, P. , van der Grinten, C. , Gustafsson, P. , Jensen, R. , Johnson, D. C. , MacIntyre, N. , McKay, R. , Navajas, D. , Pedersen, O. F. , Pellegrino, R. , Viegi, G. , Wanger, J. , & ATS/ERS Task Force . (2005). Standardisation of spirometry. The European Respiratory Journal, 26, 319–338. 16055882 10.1183/09031936.05.00034805

[phy215614-bib-0026] Moreno, R. H. , Hogg, J. C. , & Pare, P. D. (1986). Mechanics of airway narrowing. The American Review of Respiratory Disease, 133(6), 1–13. 3717766 10.1164/arrd.1986.133.6.1171

[phy215614-bib-0027] Muñoz, P. A. , Gómez, F. P. , Manrique, H. A. , Roca, J. , Barberà, J. A. , Young, I. H. , Anderson, S. D. , & Rodríguez‐Roisin, R. (2008). Pulmonary gas exchange response to exercise‐ and mannitol‐induced bronchoconstriction in mild asthma. Journal of Applied Physiology, 105(5), 1477–1485. 18756011 10.1152/japplphysiol.00108.2008

[phy215614-bib-0028] O'Donnel, C. R. , Castile, R. G. , & Mead, J. (1986). Changes in flow‐volume curve configuration with bronchoconstriction and bronchodilation. Journal of Applied Physiology, 61(6), 2243–2251. 3804929 10.1152/jappl.1986.61.6.2243

[phy215614-bib-0029] Ohwada, A. , & Takahashi, K. (2012). Concave pattern of a maximal expiratory flow‐volume curve: A sign of airflow limitation in adult bronchial asthma. Pulmonary medicine, 797495. 23227333 10.1155/2012/797495 PMC3514841

[phy215614-bib-0030] Parsons, J. P. , Hallstrand, T. S. , Mastronarde, J. G. , Kaminsky, D. A. , Rundell, K. W. , Hull, J. H. , Storms, W. W. , Weiler, J. M. , Cheek, F. M. , Wilson, K. C. , & Anderson, S. D. (2013). An official American thoracic society clinical practice guideline: Exercise‐induced bronchoconstriction. American Journal of Respiratory and Critical Care Medicine, 187(9), 1016–1027. 23634861 10.1164/rccm.201303-0437ST

[phy215614-bib-0031] Patel, A. C. , van Natta, M. L. , Tonascia, J. , Wise, R. A. , & Strunk, R. C. (2008). Effects of time, albuterol, and budesonide on the shape of the flow‐volume loop in children with asthma. The Journal of Allergy and Clinical Immunology, 122(4), 781–787. 19014770 10.1016/j.jaci.2008.08.010 PMC2881659

[phy215614-bib-0032] Pride, N. B. , & Macklem, P. T. (1985). Lung mechanics in disease. In A. P. Fishman (Ed.), Handbook of physiology: The respiratory system, section 3. Williams and Wilkins Co ch. 37.

[phy215614-bib-0033] Quanjer, P. H. , Stanojevic, S. , Cole, T. J. , Baur, X. , Hall, G. L. , Culver, B. H. , Enright, P. L. , Hankinson, J. L. , Ip, M. S. M. , Zheng, J. , Stocks, J. , & the ERS Global Lung Function Initiative . (2012). Multi‐ethnic reference values for spirometry for the 3‐95‐yr age range: The global lung function 2012 equations. European Respiratory Journal, 40(6), 1324–1343. 22743675 10.1183/09031936.00080312 PMC3786581

[phy215614-bib-0034] Rossman, M. J. , Petrics, G. , Klansky, A. , Craig, K. , Irvin, C. G. , & Haverkamp, H. C. (2022). Exercise‐induced bronchodilation equalizes exercise ventilatory mechanics despite variable baseline airway function in asthma. Medicine and Science in Sports and Exercise, 54(2), 258–266. 34559730 10.1249/MSS.0000000000002793 PMC8892975

[phy215614-bib-0035] Samee, S. , Altes, T. , Powers, P. , de Lange, E. E. , Knight‐Scott, J. , Rakes, G. , Mugler, J. P., III , Ciambotti, J. M. , Alford, B. A. , Brookeman, J. R. , & Platts‐Mills, T. A. E. (2003). Imaging the lungs in asthmatic patients by using hyperpolarized helium‐3 magnetic resonance: Assessment of response to methacholine and exercise challenge. The Journal of Allergy and Clinical Immunology, 111(6), 1205–1211. 12789218 10.1067/mai.2003.1544

[phy215614-bib-0036] Spycher, B. , Frey, U. , Wildhaber, J. H. , & Sznitman, J. (2012). Mathematical behavior of MEFV curves in childhood asthma and the role of curvature in quantifying flow obstruction. ISRN Pulmonology ID 305176. 10.5402/2012/305176

[phy215614-bib-0037] Standardization of Spirometry, 1994 Update . (1995). American Thoracic Society. American Journal of Respiratory and Critical Care Medicine, 152(3), 1107–1136. 7663792 10.1164/ajrccm.152.3.7663792

[phy215614-bib-0038] Staub, N. (1974). Pulmonary edema. Physiological Reviews, 54(3), 678–794. 4601625 10.1152/physrev.1974.54.3.678

[phy215614-bib-0039] Staub, N. C. , Nagano, H. , & Pearce, M. L. (1967). Pulmonary edema in dogs, especially the sequence of fluid accumulation in lungs. Journal of Applied Physiology, 22(2), 227–240. 6017888 10.1152/jappl.1967.22.2.227

[phy215614-bib-0040] Venegas, J. G. , Winkler, T. , Musch, G. , Melo, M. F. V. , Layfield, D. , Tgavalekos, N. , Fischman, A. J. , Callahan, R. J. , Bellani, G. , & Scott Harris, R. (2005). Self‐organized patchiness in asthma as a prelude to catastrophic shifts. Nature, 434(7034), 777–782. 15772676 10.1038/nature03490

[phy215614-bib-0041] Yager, D. , Butler, J. P. , Bastacky, J. , Israel, E. , Smith, G. , & Drazen, J. M. (1989). Amplification of airway constriction due to liquid filling of airway interstices. Journal of Applied Physiology, 66(6), 2873–2884. 2745353 10.1152/jappl.1989.66.6.2873

